# Clinical Remarks upon the Treatment of Syphilis

**Published:** 1873-05

**Authors:** Willard Parker


### Clinical Remarks upon the Treatment of Syphilis.

By Professor WILLARD PARKER, M. D.

This man whom you now see is suffering from tertiary syphilis, and that is the cause of this diseased bone upon his skull.

In connection with this case I will say a few words concerning the treatment of syphilis.

It generally occurs in those men who are addicted to the use of alcoholic drinks. It may be in the gentleman who takes his bottle of wine at dinner, or his hot drink at bedtime; who lives pretty thoroughly upon his tobacco, and then the third partner in the firm comes in in the shape of syphilis. The trio consists of rum, tobacco and syphilis.

A vast number of our men who undertake to treat syphilis, treat the syphilis alone, never thinking to break off the rum and tobacco. The patient can never get well under such circumstances With these three enemies in the system what chance can the doctor have to rout one while the other two stand ready to neutralize his efforts by carrying in sustenance for the third? I make it my rule, never to undertake the treatment of a case of syphilis unless the patient will give up his tobacco and rum, and co-operate unreservedly with me in the management of his case, and then it is comparatively an easy matter.

This is a practical point worth abiding by. Again, whenever a man has a constitutional disease, whether scrofula or syphilis or tuberculosis, or other disease, that man's system is *below par;* it is never up to the getting-well point. The first great step then is to try and bring them up to that point. Syphilis demands the quiet effects of mercury. I am aware that I differ with many of my brethren in the treatment of syphilis, but I believe that the poison of syphilis can only be removed from the system in almost all cases by the judicious and wise use of mercury. This mercury is to be used wisely and in moderate doses, so as not to impair the vigor and health of the system. Very often it is important to make use of some tonic at the same time, such as quinine or the preparations of bark. These have been my convictions for a great many years, and I give them as the result of my own practical observation, and have never seen any reason to vary the conviction, that iodide of potassium alone cannot overcome the syphilitic poison in the system. The iodide of potassium, however, is a very valuable remedy in treatment of syphilis, but it comes in after we have accomplished our purpose with mercury, in order to remove any deleterious effects of mercury which may be left in the system. Here its value cannot be overestimated. The powerful effect which the iodide of potassium has upon the system, especially where mercury has been employed pretty freely, is sometimes seen in the profuse ptyalism which it produces, and if the syphilis receives any benefit from the administration of the iodide of potassium, I believe it is in those cases whioh have heretofore been treated with mercury and the iodide arouses the mercury to new action. You can remove mercury from the system by the use of iodide of potassium, but you can never remove syphilis by using it. At the same time we use iodide of potassium in order to get good results in the system, I almost always employ the iodide of iron, as you see in this case. The point is, as has been stated, to bring the system up to par. The usual formula which I employ consists in six drachms of the iodide of potassium, one ounce of syrup iodide of iron, and make a six or eight ounce mixture. When this man first came here he was exsanguineous, and had a hang-dog look, but now his face looks ruddy and his system is fast getting into the proper condition for the manufacture of good blood. His system is being brought up, and now if injures himself in any way, proper reparative processes will at once be instituted. The plan which I adopt and recommend in the treatment of syphilis is as follows: Take a case of genuine Hunterian chancre. I commence with the administration of iodide of mercury in one-half grain doses twice in twenty-four hours, combined with something, perhaps hyoscyamus or lactucarium, to prevent irritation of the mucous membrane of the intestinal canal. Continue this, in connection with a true diet, consisting of simple plain material and such as will produce healthy blood, embracing breadstuffs, eggs, milk and meat twice a day, and, cutting off entirely tobacco and all alcoholic drinks, continue the doses until the feeling of hardness about the chancre is all gone. Then stop the remedy, and watch the patient. If the disease begins to come out in the system, manifesting itself by glandular enlargements, diseases of the skin, affections of the fauces, or any one of these evidences, which shows that the poison is still in the system, resume the mercury as before and continue it until the disease has again passed away. It will be necessary to watch these patients for a long time, at least for months, and perhaps for a couple of years or more.—*Medical Record.*

